# Cheiloplasty

**Published:** 1856-01

**Authors:** 


*Cheiloplasty.*—This operation of lip-making has been performed by Professor Eve, of Nashville, in a case of great difficulty. The patient was a stout healthy youth of good habits, aged 18 years. The upper lip had been destroyed by salivation. From the points of each commissure of the mouth upwards for about three-fourths of an inch, and across the base of the septum of the nose, including one of its wings, and a portion of its column, was a vacant space occupied only by the teeth and gums. A hard cicatrix defined the outline, closely attached to the alveolar and bony structures. This was exsected, and then two long incisions, about one inch apart, on each side, were made through the cheek, extending to near the ears. These were dissected up to make a new lip, and secured with three hare-lip pins and one suture in the mesial line. Stitches were placed in the cheeks, and the whole was further secured by adhesive strips, compresses and rollers. The operation was successful, presenting "a pretty fair upper lip;" and is the most extensive and difficult operation of the kind which has ever been performed, constituting a triumph of surgical skill highly creditable to the distinguished operator.—*Memphis Recor.*

